# Absence of PEXEL-Dependent Protein Export in *Plasmodium* Liver Stages Cannot Be Restored by Gain of the HSP101 Protein Translocon ATPase

**DOI:** 10.3389/fgene.2021.742153

**Published:** 2021-12-08

**Authors:** Oriana Kreutzfeld, Josephine Grützke, Alyssa Ingmundson, Katja Müller, Kai Matuschewski

**Affiliations:** ^1^ Molecular Parasitology, Institute of Biology/Faculty for Life Sciences, Humboldt Universität zu Berlin, Berlin, Germany; ^2^ Parasitology Unit, Max Planck Institute for Infection Biology, Berlin, Germany; ^3^ Department of Medicine, University of California, San Francisco, San Francisco, CA, United States; ^4^ Department of Biological Safety, Federal Institute for Risk Assessment, Berlin, Germany

**Keywords:** malaria, plasmodium, protein export, pre-erythrocytic stage, PEXEL motif, PTEX translocon, ATPase, heat shock protein

## Abstract

Host cell remodeling is critical for successful *Plasmodium* replication inside erythrocytes and achieved by targeted export of parasite-encoded proteins. In contrast, during liver infection the malarial parasite appears to avoid protein export, perhaps to limit exposure of parasite antigens by infected liver cells. HSP101, the force-generating ATPase of the protein translocon of exported proteins (PTEX) is the only component that is switched off during early liver infection. Here, we generated transgenic *Plasmodium berghei* parasite lines that restore liver stage expression of *HSP101. HSP101* expression in infected hepatocytes was achieved by swapping the endogenous promoter with the *ptex150* promoter and by inserting an additional copy under the control of the elongation one alpha (ef*1α*) promoter. Both promoters drive constitutive and, hence, also pre-erythrocytic expression. Transgenic parasites were able to complete the life cycle, but failed to export PEXEL-proteins in early liver stages. Our results suggest that PTEX-dependent early liver stage export cannot be restored by addition of HSP101, indicative of alternative export complexes or other functions of the PTEX core complex during liver infection.

## Introduction

Host cell remodeling is critical for successful *Plasmodium* replication inside erythrocytes and achieved by targeted export of parasite-encoded proteins. Therefore, the parasite exports a large set of proteins to remodel the host erythrocyte, which is particularly important for plasma membrane fluidity and adhesive properties (Jonsdottir et al., 2021). The repertoire of exported proteins, termed exportome, is estimated to be approximately 10% of the *Plasmodium* proteome, and a quarter of the exported proteins are likely essential for parasite survival during blood stage development (Maier et al., 2008; Spielmann and Gilberger, 2015). Exported proteins have three major destinations in host erythrocytes, i) parasite-generated membranous structures, *e.g.* Maurer’s clefts (Mundwiler-Pachlatko and Beck, 2013) and caveolae-vesicle complexes (Akinyi et al., 2012), ii) the erythrocyte membrane cytoskeleton (Warncke and Beck, 2019), and iii) the erythrocyte plasma membrane (Wahlgren et al., 2017).

Export of proteins across two apposed membranes, the parasite plasma membrane and the parasitophorous vacuole membrane (PVM), requires a tightly coordinated translocation process. Similar to the classic secretory pathway, *Plasmodium* exported proteins enter the endoplasmic reticulum (ER) and are eventually secreted into the PV, although the underlying mechanism is still inadequately understood (Matthews et al., 2019a). The signature export label for this destination is a small host cell targeting (HT) sequence or *Plasmodium* export element (PEXEL), comprising the amino acid residues RxLxE/Q/D located downstream of the ER signal sequence (Hiller et al., 2004; Marti et al., 2004; Mesén-Ramírez et al., 2016). A different class of exported proteins lack this motif, so-called PEXEL/HT negative exported proteins (PNEPs), but share the downstream translocation pathway (Grüring et al., 2012; Heiber et al., 2013). Both PEXEL and PNEP proteins are believed to be loaded into secretory vesicles to be transported and released into the PV. Once in the PV, proteins need to translocate the PVM to reach the erythrocyte cytoplasm, a process mediated by the *Plasmodium* translocon of exported proteins (PTEX) (de Koning-Ward et al., 2009).

Studies in human and rodent *Plasmodium* species revealed that the PTEX complex consists of three core proteins, EXP2, HSP101, PTEX150, which are flanked by two auxiliary proteins, PTEX88 and TRX2 (de Koning-Ward et al., 2009; Matthews et al., 2013; Matz et al., 2013), as well as the GPI-anchored surface protein P113 (Elsworth et al., 2016). Additional identified auxiliary proteins include the Export Protein Interacting complex, which comprises of the parasitophorous vacuole protein 1 (PV1), PV2 and EXP3 (Batinovic et al., 2017). EXP2 plays a central role as a membrane-spanning component, but simultaneously acts as a nutrient channel in a PTEX-independent manner (Garten et al., 2018). HSP101 is considered to be the force-generating motor protein and a member of the ClpB-type AAA^+^-ATPase family (Beck et al., 2014; Matthews et al., 2019b). HSP101 contains a cargo-binding pore loop at the amino-terminal end and is most likely responsible for protein unfolding and active translocation through the complex. Although there is little evidence for a distinct functional role of PTEX150, numerous studies consistently confirmed that PTEX150 appears to play a structural role linking EXP2 and HSP101 (Bullen et al., 2012; Elsworth et al., 2014; Elsworth et al., 2016; Garten et al., 2018). Experimental genetics studies of the three core components in human and rodent *Plasmodium* species corroborated the importance of these proteins for parasite blood stage survival, and loss-of-function mutants failed to propagate during blood infection (Matthews et al., 2013; Matz et al., 2013; Beck et al., 2014; Elsworth et al., 2014). The role of PTEX88 in protein translocation is less well understood. Knockout of *PTEX88* in a murine malaria model reduced experimental cerebral malaria and cytoadherence (Matthews et al., 2013; Matz et al., 2013; Matz et al., 2015; Chisholm et al., 2016). It, thus, might play a role in translocation of specific proteins important for parasite virulence.

Although host cell remodeling and protein export are remarkably expanded in asexual blood stages, they occur both in gametocytes and during liver stage development (Ingmundson et al., 2014). This observation is further corroborated by the presence of syntenic genes in closely related *Hepatocystis* parasites, which only replicate in the liver and lack blood schizogony (Ejotre et al., 2021), yet harbor orthologous genes for *EXP2* (HEP_00110700), *HSP101* (HEP_00056400), *PTEX150* (HEP_ 00473300) and *PTEX88* (HEP_004505500) (Aunin et al., 2020). One hypothesis is that during liver infection the malarial parasite appears to curtail protein export. As metabolically active cells and in contrast to red blood cells, hepatocytes permit the developing parasite to exploit the host cell, likely limiting the need for intensive remodeling and, thereby, directing energy towards population expansion (Prudencio et al., 2006; Silvie et al., 2008a).

Moreover, hepatocytes harbor an active MHCI pathway with the potential to present peptide fragments of intracellular parasitic antigens to the host’s immune cells (Chakravarty et al., 2007). To avoid recognition by the host, the parasite might keep protein translocation during early to mid liver stage development to a minimum. To date, only peptides of sporozoite origin could be identified as targets of CD8^+^ T cells (Bongfen et al., 2007; Hafalla et al., 2013; Müller et al., 2017). Recently, a liver stage specific peptide, Kb-17, was identified, but its contribution to protective immune responses remains to be determined (Pichugin et al., 2018).

To date, three proteins were reported to translocate during liver stage growth, namely circumsporozoite protein (CSP) (Singh et al., 2007), liver-specific protein 2 (LISP2) (Orito et al., 2013), and sporozoite- and liver stage-expressed tryptophan rich protein (SLTRiP) (Jaijyan et al., 2015). The extent of CSP export in infected hepatocytes remains highly controversial (Singh et al., 2007; Cockburn et al., 2011), partly because CSP might be already released into the host’s cytoplasm during sporozoite cell traversal and upon hepatocyte entry. Immunofluorescence assays with LISP2 antibodies demonstrated onset of export 24 h after infection, yet parasites harboring a C-terminal mCherry tagged LISP2 failed to export the tagged protein to the hepatocyte cytoplasm (Orito, et al., 2013). SLTRiP export in liver stages was detected as early as 12 h after infection, with 80% of infected host cells showing export of SLTRiP (Jaijyan et al., 2015). A central question is whether a potential liver stage exportome is translocated by similar or distinct mechanisms as compared to translocation during blood infection.

The notion of very restricted protein export during the first schizogony in infected hepatocytes is supported by additional experimental genetics evidence. Fusion of the PEXEL motif of *Plasmodium berghei* CSP to the model antigen ovalbumin and expression under the control of the *UIS4* promoter failed to induce export in infections with transgenic parasites, although CD8^+^ T cell proliferation was increased (Montagna et al., 2014). Moreover, during liver stage maturation HSP101 is not expressed until 48 h after infection, whereas all other PTEX components are present (Matz et al., 2015), offering a plausible explanation for restricted protein export in these stages. Investigation of KAHRP_L_-GFP PEXEL dependent export in liver stages showed absence of such export despite the presence of EXP2 and PTEX150. Further experiments with HA-tagged HSP101 parasites confirmed the absence of HSP101 in liver stages, an indicator for lack of PEXEL dependent export in liver stages (Kalanon et al., 2016). A valid hypothesis, experimentally addressed in the present study, is that the *Plasmodium* PTEX ATPase HSP101 might be a limiting factor for liver stage protein export. Alternatively, the protein unfolding and translocation functions of HSP101 might be substituted by a distinct, yet unidentified factor.

An important implication is whether increasing antigen export in early liver stages would result in increased immune recognition of *Plasmodium*-infected cells, by presentation of parasite-derived epitopes *via* MHCI. We, therefore, sought to define liver stage export in *HSP101* over-expressing parasites and to study these parasite lines for potential enhanced CD8^+^ T cell responses and protection in cell-based immunization.

To this end, we designed *P. berghei* lines that express *HSP101* (PBANKA_0931200) during liver stage maturation. This gain-of-function approach is expected to allow PTEX reconstitution, since the other major PTEX components, PTEX150 and EXP2, and the auxiliary factor PTEX88 are expressed during liver stage development (Matz et al., 2015). Reconstitution of the PTEX export machinery might facilitate protein export in early liver stages and, thus, might promote antigen presentation and recognition of parasites by the host immune system.

## Results

### Defined PEXEL Regions Lead to Export of mCherry Reporter Constructs in the Blood Stage, but not in the Liver Stage

**FIGURE 1:**
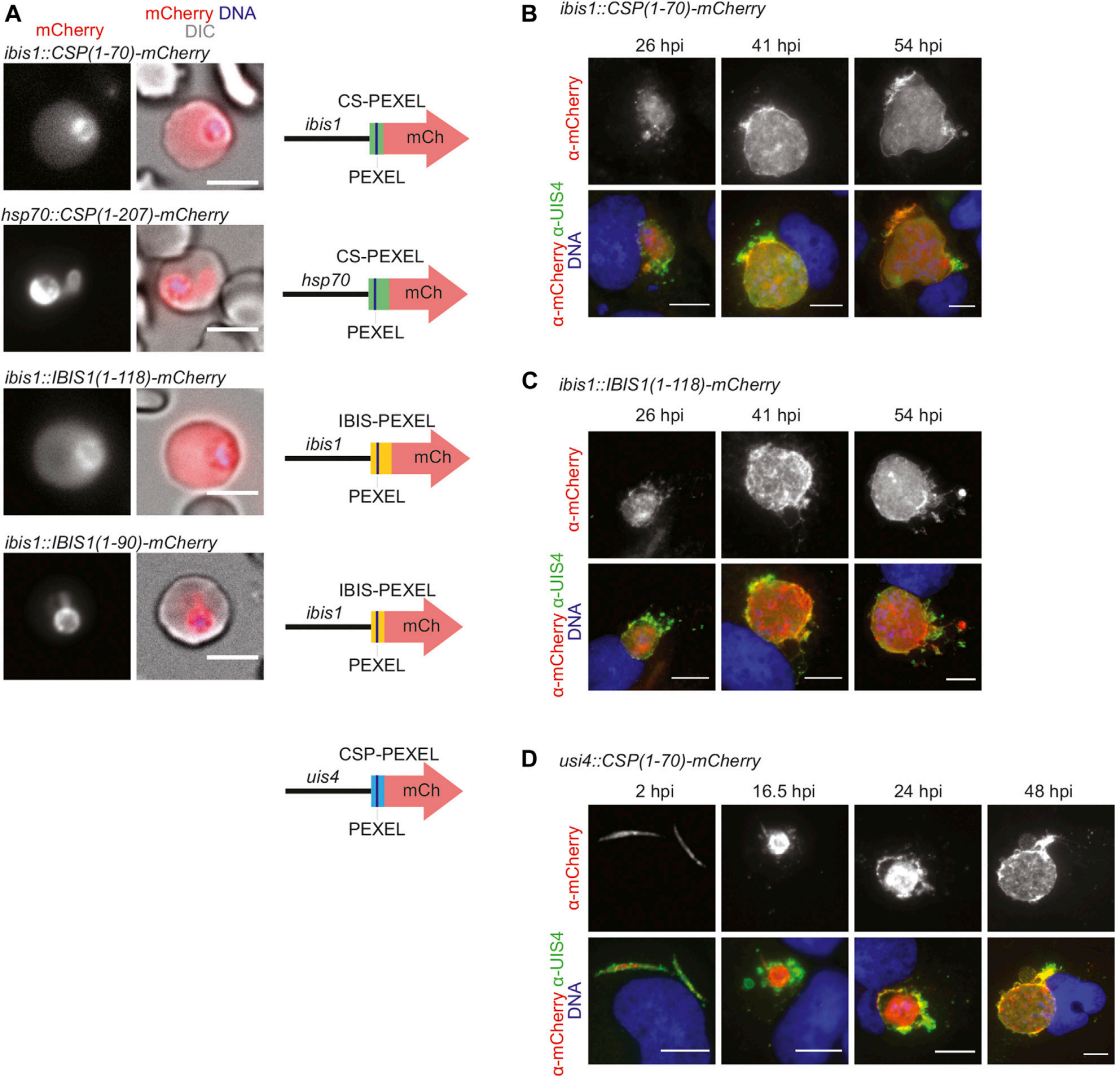
PEXEL-dependent export of mCherry reporter protein occurs during blood but not liver infection. **(A)** Live fluorescence imaging of parasite-infected erythrocytes. Shown are the mCherry signal **(left)**, a merge with DIC images **(center)**, and graphic images of the reporter constructs **(right).** Depicted are promoters, PEXEL regions (green, yellow, blue), the PEXEL motif (line), and the mCherry open reading frame (red arrow). Top row, erythrocyte infected with a transgenic *CSP_1-70_-mCherry* parasite under the control of the *IBIS1* promoter. Upper center row, erythrocyte infected with a transgenic *CSP_1-70_-mCherry* parasite under the control of the *hsp70* promoter. Lower center row, erythrocyte infected with a transgenic *IBIS_1-118_-mCherry* parasite under the control of the *IBIS1* promoter. Bottom row, erythrocyte infected with a transgenic *IBIS_1-90_-mCherry* parasite under the control of the *IBIS1* promoter. Bar, 5 μm. **(B–D)** Immunofluorescent staining of Huh7 cells infected with the parasite lines expressing either **(B)** *CSP_1-70_-mCherry*, **(C)** *IBIS1_11-118_-mCherry*, or **(D)** *CSP-PEXEL-mCherry* under the control of the promoter indicated and at the indicated time points after sporozoite infections. Shown are *α*-mCherry signals **(top row)** and merge images **(bottom row)** of *α*-mCherry (red), *α*-UIS4 (green), and Hoechst 33,342 (blue) stain. Bar, 10 µm.

To compare PEXEL-dependent export in *P. berghei* blood and liver stages we generated several reporter lines, which encode mCherry fusion proteins that contain predicted *CSP-PEXEL* and *IBIS-PEXEL* motifs at their amino-terminal end (Figure 1A; Supplementary Figure S1). Export of the fluorescent reporter proteins into red blood cells was analyzed by live immunofluorescence imaging. The first set of transgenic parasite lines expressed the PEXEL-containing reporter proteins under the control of the *IBIS1* promoter, which is active in several life cycle stages, including in liver and blood stages. Reporter proteins that included additional amino acids after the RxLxQ/E/D motif, CSP_1-70_-mCherry and IBIS_1-118_-mCherry, were exported to the infected erythrocytes (Figure 1A). When a truncated version of an IBIS1 fusion protein, IBIS1_1-90_-mCherry, was expressed under the control of the *IBIS1* promoter, the red fluorescent signal was confined to the parasite and displayed an enrichment at the PV. A similar confinement to the parasite and a stronger signal at the PV was detected for transgenic parasites that expressed CSP_1-70_-mCherry under the control of the strong and ubiquitous *HSP70* promoter (Figure 1A), indicative that excessive protein may congest the PTEX translocon.

We wanted to determine the fate of the two fusion proteins, CSP_1-70_-mCherry and IBIS_1-118_-mCherry, that were exported during blood infection, in liver stages. When hepatoma cells were infected with the lines described above that express these proteins from the *IBIS1* promoter, mCherry remained confined within the liver-stage PVM (Figures 1B,C). Because *IBIS1* is not expressed until the mid-liver stage (Ingmundson et al., 2012), we generated an additional transgenic line in which CSP_1-70_-mCherry is under the control of the *UIS4* promoter to examine the localization of the reporter early in the pre-erythrocytic stage (Silvie et al., 2014). Fluorescent imaging of hepatoma cells infected with these transgenic sporozoites revealed expression of mCherry as early as 2 h after infection and throughout liver stage maturation up until 48 h later, but the signal remained within the membranes of the parasitophorous vacuole and associated tubovesicular network (Figure 1D).

Together, PEXEL-dependent export of reporter constructs was readily detected in blood stages, but liver stage export was not observed.

### Generation of *Plasmodium berghei* Lines That Constitutively Express *HSP101*

To test whether PEXEL-dependent protein export in *P. berghei* liver stages is limited by the absence of the PTEX component HSP101, we next generated transgenic parasite lines that express *HSP101* using two complementary strategies, i) by insertion of an additional *HSP101* copy under the control of a constitutively active promoter, and ii) by swapping the endogenous promoter with a constitutive promoter. Both strategies are predicted to allow full reconstitution of a functional PTEX in liver stages, since PTEX150, EXP2, and PTEX88 are expressed during liver stage maturation (Matz et al., 2015).

Plasmids for *P. berghei* transfection were assembled in the pBAT and the pBART-SIL6 vector (Kooij et al., 2012). These targeting vectors harbor the drug-selectable *hDHFR-yFcu* cassette for positive/negative selection and a GFP expression cassette. The latter is used to acquire isogenic lines by fluorescence-activated cell sorting (FACS) and for live imaging of the parasite cytoplasm during the entire parasite life cycle. The pBART-SIL6 vector also contains two homologous regions to integrate into a silent region of chromosome 6 (SIL6) (Supplementary Figure S2).

We generated two targeting plasmids, which express an additional copy *P. berghei HSP101* (*Pb*ANKA_0931200) under the control of the constitutive promoter of elongation factor 1*α* (*ef1*α, PBANKA_113330) either as a fusion protein with a triple Myc tag only (*ef1α:HSP101-myc*) or an additional mCherry tag (*ef1α:HSP101-mCherry*) (Supplementary Figure S3A). Upon transfection and positive selection transgenic parasites were readily obtained and labeled *ef1α:HSP101-myc* or *-mCherry,* respectively. As a complementary approach, we replaced the endogenous promoter with the promoter of PTEX150 (PBANKA_1008500), which is expressed during liver stage development (Matz et al., 2015) and is expected to permit the appropriate temporal expression of *HSP101* (Supplementary Figure S3C). The corresponding parasite line, which was selected after transfection was labeled *HSP101:5′ptex150* to denote the promoter swap and retainment of *HSP101* at the original locus. For all three parasite lines the desired integration was confirmed by diagnostic PCR, demonstrating 5′ and 3′ integration and absence of WT parasites (Supplementary Figures S3B,D). Successful generation of the two *ef1α:HSP101* parasite lines and *HSP101:5′ptex150* parasites already indicated that additional expression from a second copy or exchange of the endogenous *HSP101* does not interfere with parasite blood stage development. *HSP101:5′ptex150* parasites harbor the endogenous *HSP101* copy under the *ptex150* promoter. As correct PTEX assembly is vital for parasite survival (Matthews et al., 2013; Matz et al., 2013; Beck et al., 2014; Elsworth et al., 2014), the ability to generate and grow *HSP101:5′ptex150* parasites indicated that correct PTEX assembly was achieved with *HSP101* expressed under the *ptex150* promoter. Hence, we could characterize life cycle progression and analyze protein export during parasite maturation in the liver.

We next examined life cycle progression of the transgenic parasite lines. *ef1α:HSP101-mCherry* blood stages developed normally and displayed an mCherry signal consistent with PV localization (Supplementary Figures S4A,B), indicative of proper localization of the HSP101-mCherry fusion protein. Female *Anopheles stephensi* mosquitoes were allowed to feed on mice infected with *ef1α:HSP101-mCherry* parasites. Successful development in the mosquito was determined on day 10 for oocysts, day 14 for midgut sporozoites and day 17 for salivary gland sporozoite formation and results were compared to *P. berghei* WT parasites (*bergreen*). *ef1α:HSP101-mCherry*-infected mosquitoes showed normal oocyst and sporozoite development, and HSP101-mCherry was moderately expressed in ef*1α:HSP101-mCherry* oocysts and sporozoites (Supplementary Figure S4C). Midgut infectivity and sporozoite numbers in freshly dissected salivary glands of all three transgenic lines were similar to WT (Supplementary Table S1).

Overall, transgenic parasites underwent normal life cycle progression enabling analysis of liver stage development and protein export.

### Ectopically Expressed HSP101 Localizes to the Parasitophorous Vacuole in Liver Stages

**FIGURE 2:**
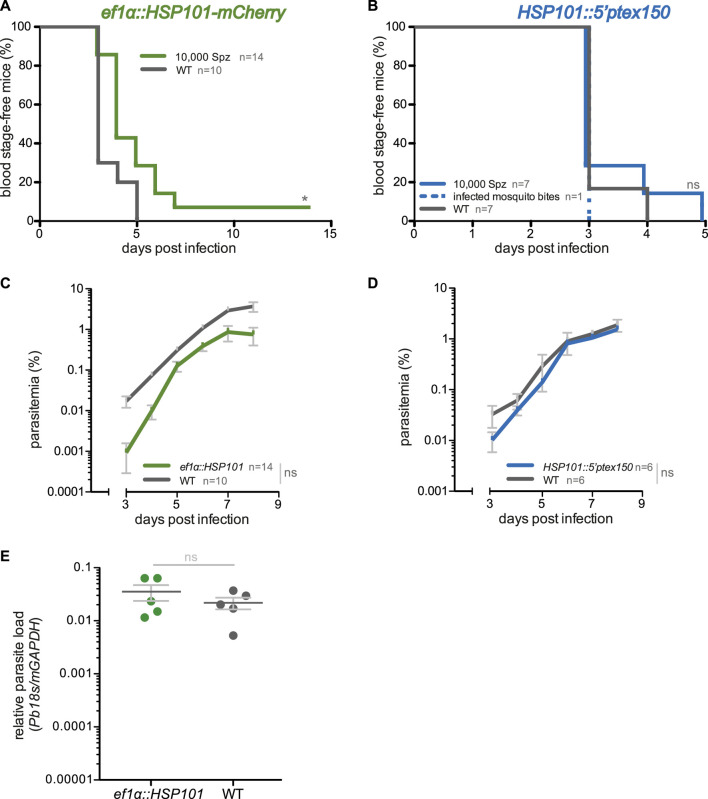
Blood infections induced by *ef1α:HSP101-mCherry* and *hsp101:5′ptex150* sporozoites. **(A,B)** Shown are Kaplan-Meier analyses of blood infection over time after intravenous injection of 10,000 sporozoites isolated from **(A)** *ef1α:HSP101-mcherry* or **(B)** *hsp101:5′ptex150*-infected *Anopheles stephensi* mosquitoes. Exposure to 15 *hsp101:5′ptex150*-infected mosquitoes is included in **(B)**. **(C,D)** Blood stage growth in **(C)** *ef1α:HSP101-mCherry* or **(D)** *hsp101:5′ptex150*-infected mice. **(E)** qPCR analysis of relative parasite liver load 48 h after sporozoite infection. Shown are mean values (±SD). Blood infection was monitored daily by microscopic examination of Giemsa-stained blood films. *, *p* < 0.05; n. s., non-significant (Log rank test for Kaplan-Meier analysis and slope analysis; Mann-Whitney-U test for relative parasite liver load).

We first examined whether *ef1α:HSP101-mCherry* and *HSP101:5′ptex150* sporozoites are able to undergo pre-erythrocytic development and establish a patent blood infection. Groups of C57BL/6 mice were infected intravenously with 10,000 of WT, *ef1α:HSP101-mCherry* or *HSP101:5′ptex150* sporozoites and monitored for the time to occurrence of the first blood stage parasite, termed pre-patency, and parasite growth dynamics (Figure 2). We noticed a slight delay of approximately 1 day in pre-patency in mice infected with *ef1α:HSP101-mCherry* parasites in comparison to WT (Figure 2A). The delayed onset of blood infection was clearly visible in the analysis of blood stage propagation (Figure 2C).

We independently confirmed this finding by intravenous injection of the second transgenic line, *ef1α:HSP101-myc*, which also displayed a 1 day delay in patency (Supplementary Figure S5). But exposure to 15 *ef1α:HSP101-myc*-infected mosquitoes resulted in a normal prepatent period of 3 days (Supplementary Figure S5). This finding prompted us to quantify the parasite RNA levels in livers infected with mature liver stages, *i.e.* 48 h after sporozoite inoculation (Figure 2E). In this analysis, no significant differences were detectable between *ef1α:HSP101-mCherry* and WT-infected mice, indicating that the observed delay is not due to slower liver stage maturation. Remarkably, and in contrast to *ef1α:HSP101-mCherry* infections, there was no difference in *HSP101:5′ptex150* sporozoites parasite growth as compared to WT (Figures 2B,D).

**FIGURE 3:**
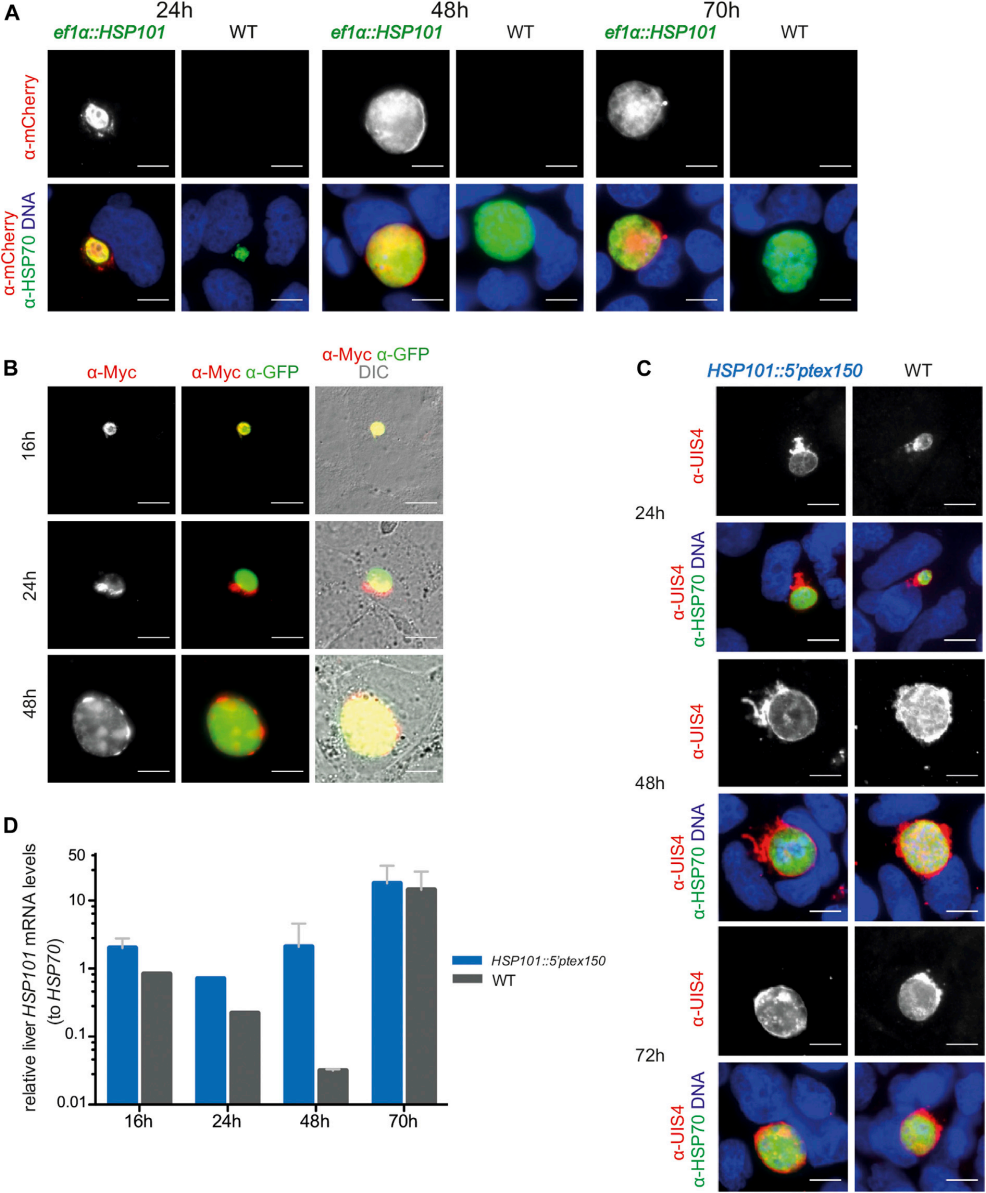
Ectopic expression of HSP101 in liver stages results in PV localization. **(A)** Localization of HSP101-mCherry in *ef1α:HSP101-mCherry*-infected hepatoma cells at 24, 48, and 72 h after infection. Shown are immunofluorescent images with *α*-mCherry antibody **(top row)** and merge images **(bottom row)** displaying the signals of *α*-mCherry (red), *α*-HSP70 (green), and Hoechst 33,342 (blue). Bars, 10 µm. **(B)** Localization of HSP101-myc in *ef1α:HSP101-myc*-infected hepatoma cells at 16, 24, 48 h after infection. Shown are immunofluorescent images with an *α*-myc antibody **(left column)**, *α*-myc (red) and *α*-GFP (green) antibodies **(center column)**, and merge images **(right column)** displaying the signals of *α*-myc (red), *α*-GFP (green), and DIC. Bars, 10 µm. **(C)** Liver stage development of *hsp101:5′ptex150* parasites at 24, 48, and 72 h after infection. Shown are immunofluorescent images with an *α*-UIS4 antibody **(top)**, and merge images **(bottom)** displaying the signals of *α*-UIS4 (red), *α*-HSP70 (green), and Hoechst 33,342 (blue). Bars, 10 µm. **(D)** Steady state *HSP101* mRNA levels in *hsp101:5′ptex150* liver stages. Shown are relative mRNA levels determined qRT-PCR from total RNA of *hsp101:5′ptex150* or WT-infected hepatoma cells at indicated time points after infection. ∆∆CT values of *HSP101* mRNA values were normalized to *HSP70*. Depicted are mean values (±SD) (n = 2, except for 24h, n = 1).

We infected hepatoma cells with freshly dissected salivary gland sporozoites and monitored liver stage size, morphology, HSP101 expression, and numbers of infected cells (Figure 3). The sizes of liver stages were similar for all transgenic lines and WT parasites. In *ef1α:HSP101-mCherry* parasites successful expression of HSP101 could be detected, using the red mCherry and myc tags as indicator. HSP101-mCherry localized to the liver stage PVM (Figure 3A). This localization was independently confirmed in hepatoma cells infected with *ef1α:HSP101*-myc sporozoites (Figure 3B). For *HSP101:5′ptex150* parasites growth was monitored by immunofluorescence analysis (Figure 3C). All three transgenic lines showed similar growth rates compared to WT (Figure 3).

In contrast to *ef1α:HSP101-mCherry* parasites, HSP101 expression in *HSP101:5′ptex150* could not be verified by immunofluorescence, since the endogenous open reading frame was retained. As a proxy for expression, we determined steady-state *HSP101* mRNA levels (Figure 3D). As intended, *HSP101* mRNA levels were significantly elevated at 24 and 48 h in *HSP101:5′ptex150* liver stages.

In conclusion, gain of *HSP101* expression resulted in successful liver stage development and HSP101 localization to the PVM in infected liver cells. While onset of blood infection was delayed in *ef1α:HSP101-mCherry* parasites, *HSP101:5′ptex150* infections developed parasite infections indistinguishable from WT.

### Absence of PEXEL-Dependent Export in *ef1α:HSP101-mCherry* and *HSP101:5′ptex150* Liver Stages

To determine whether *ef1α:HSP101-mCherry* or *HSP101:5′ptex150* parasites display export of PEXEL-containing proteins during early liver stage development, we performed genetic crosses of the three transgenic parasite lines with four parasite lines that express reporter proteins (Table 1). Three of the lines express reporter proteins that were successfully exported during blood infection, namely *uis4:CSP*_*1-70*_ *-mCherry, uis4:IBIS*_*1-118*_ *-mCherry,* and *ibis1:IBIS*_*1-118*_ *-mCherry* (Figure 1), and one line, *uis4:CSP*_*1-70*_ *-OVA,* expresses the model antigen ovalbumin fused to the predicted CSP PEXEL motif (Montagna et al., 2014), which is also predicted to be exported in the blood stage*.* Genetic crosses were obtained by feeding *Anopheles stephensi* mosquitoes on mice, which were double infected with either *ef1α:HSP101-myc* or *HSP101:5′ptex150* parasites and the export reporter line (Supplementary Figure S6). Sporozoites were then isolated for hepatoma cell infection.

**TABLE 1:** Export ability of reporter lines in transgenic HSP101-expressing liver stages.

Transgenic parasites	Reporter line	Export (Yes/No)
*ef1α:HSP101-mCherry*	*uis4:CS-PEXEL-OVA*	*N*
*ef1α:HSP101-myc*	*uis4:CS-PEXEL-mCherry*	*N*
	*uis4:IBIS1-PEXEL-mCherry*	*N*
	*ibis1:IBIS1-PEXEL-mCherry*	*N*
	*uis4:CS-PEXEL-OVA*	*N*
*HSP101:5′ptex150*	*uis4:CS-PEXEL-mCherry*	*N*
	*uis4:IBIS1-PEXEL-mCherry*	*N*
	*ibis1:IBIS1-PEXEL-mCherry*	*N*
	*uis4:CS-PEXEL-OVA*	*N*

**FIGURE 4:**
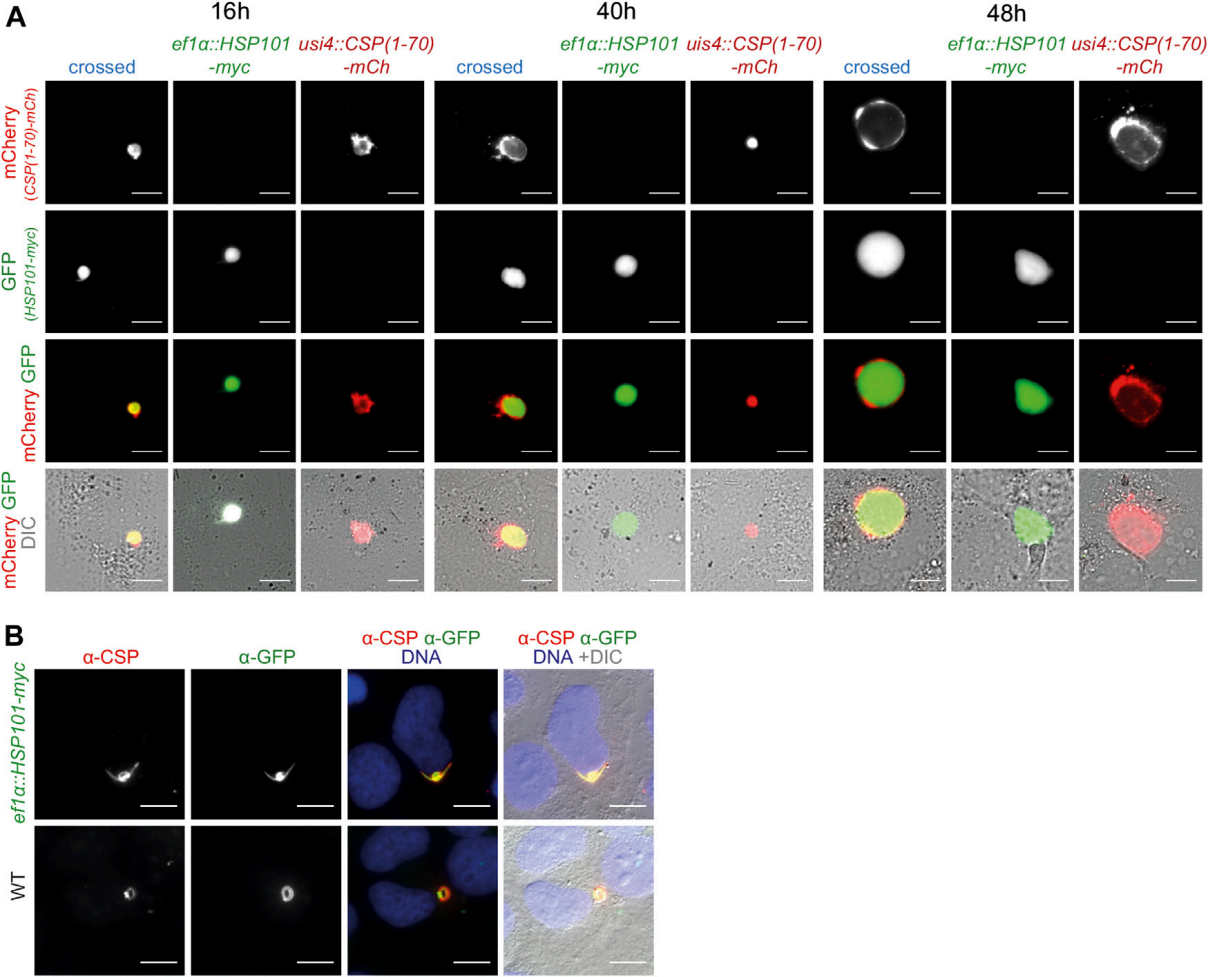
Ecctopic expression of HSP101 in liver stages results in PV localization. **(A)** Live imaging of hepatoma cells infected with sporozoites from a genetic cross of *ef1α:HSP101-myc* and *UIS4:CSP-PEXEL-mCherry* (crossed, blue), and the two individual strains, *ef1α:HSP101-myc* (green), *UIS4:CSP-PEXEL-mCherry* (red). Shown are the PEXEL model protein fused to mCherry **(top row,** red), cytoplasmic GFP of the *ef1α:HSP101-myc* parasite **(upper center row,** green), the merge of both signals **(lower center row)**, and a merge of both fluorescent signals with DIC images **(bottom row)**. Note that the mCherry-tagged model antigen remains confined to the PV in HSP101-expressing parasites. Bars, 10 µm. **(B)** Immunofluorescence analysis of CSP in *ef1α:HSP101-myc* **(top row)** and WT **(bottom row)** parasites, 4 h after infection. Shown are the signals of *α*-CSP **(left)** and *α*-GFP **(centre left)**, the merge of both signals together with Hoechst 33,342 (blue; **centre right)**, and a merge of all fluorescent signals with DIC images **(right)**. Bars, 10 µm.

After sexual recombination in the brief phase of the diploid/tetraploid zygote of the otherwise haploid parasite life cycle a quarter of the mixed sporozoite population carries both desired genetic modification, since they are coded on different chromosomes. They can be directly visualized by fluorescent imaging, with GFP only in *HSP101* expressing parasites and mCherry or ovalbumin in the reporter constructs. We evaluated protein export in liver stages by both, live imaging and after antibody staining. As exemplified for one cross, *ef1α:HSP101-myc* and *UIS4:CSP*_*1-70*_ *-mCherry,* the tagged protein stayed confined to the PV at all time points during liver stage maturation, similar to the reporter line in a WT background (Figure 4A). We systematically determined protein export in all nine crosses and noted similar observations of no indication for export into the host hepatocyte. We independently tested the distribution of CSP (Singh, et al., 2007; Cockburn, et al., 2011) by antibody staining. Even in the presence of HSP101 we could not capture CSP signal outside the area of the *ef1α:HSP101-myc* parasite (Figure 4B).

Thus, protein export could not be induced in *ef1α:HSP101-myc* or *HSP101:5′ptex150* parasites, despite a range of PEXEL sequences and assessment of different time points during liver stage maturation. Accordingly, HSP101 is not a limiting factor for PEXEL-dependent protein export during this life cycle stage.

### Attenuated *ef1α:HSP101-mCherry* and *HSP101:5′ptex150* Sporozoites Elicit Weak Vaccine-Induced Protection

**FIGURE 5:**
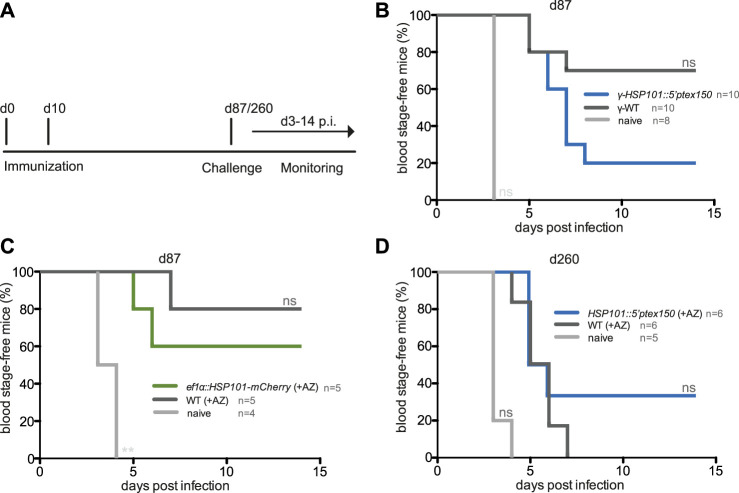
Inferior protection in mice vaccinated with attenuated *ef1α:HSP101-mCherry* or *HSP101:5′ptex150* sporozoites. **(A)** Overview of immunization and challenge protocol. C57bl/6 mice were immunized twice at a 10-days interval with 10,000 intravenously injected (i.v.) sporozoites. Challenge infections with 10,000 i. v. WT sporozoites was done at indicated time points. Monitoring of blood infection was done daily by microscopic examination of Giemsa-stained blood films. **(B**–**D)** Kaplan-Meier analysis of blood infection over time after intravenous sporozoite injection. **(B)** Immunizations with irradiated *HSP101:5′ptex150* (blue) and WT (black) sporozoites. **(C)** Immunizations with azithromycin-arrested *ef1α:HSP101-mCherry* (green) and WT (black) sporozoites. **(B,C)** Challenge infections were done on day 87 (77 days after the booster immunization). **(D)** Immunizations with azithromycin-arrested *HSP101:5′ptex150* (blue) and WT (black) sporozoites. Challenge infections were done on day 260 (250 days after the booster immunization). Naive mice (grey) served as infection controls in all experiments. n. s, non-significant (Log rank (Mantel-Cox) test).

We finally wanted to explore whether gain of HSP101 function modulated vaccine-induced protection. One hypothesis is that a larger repertoire of yet unidentified parasite antigens might increase immunogenicity during liver stage development, and processing of exported parasite proteins might be an important contributing factor. To test this notion, protective efficacy of immunization with live attenuated *ef1α:HSP101-mCherry* or *HSP101:5′ptex150* parasites was compared to WT immunizations (Figure 5). Groups of C57BL/6 mice received a prime/boost vaccination scheme of 10,000 sporozoites, followed by azithromycin treatment to elicit late liver stage arrest (Friesen et al., 2010). Challenge was done 3 months later by intravenous injection of 10,000 WT sporozoites (Figure 5A). This protocol has consistently shown incomplete sterile protection permitting evaluation of superior or inferior vaccination schemes (Friesen and Matuschewski, 2011). *ef1α:HSP101-mCherry* and WT sporozoite -immunized mice displayed substantial sterile protection, with three and four out of five mice, respectively, blood infection-free (Figure 5B). In contrast, only two out of 10 *HSP101:5′ptex150* -immunized mice were protected in comparison to seven out of 10 in WT-immunized mice (Figure 5C). Protection in *HSP101:5′ptex150* -immunized mice under azithromycin cover vanished completely when challenged at a late time point, 260 days after immunization (Figure 5D).

Collectively, immunizations with live *ef1α:HSP101-mCherry* or *HSP101:5′ptex150* sporozoites followed by attenuation with azithromycin does not lead to superior protection, but instead results in reduced vaccine efficacy.

## Discussion

In this study, we systematically analyzed PEXEL-dependent protein export during liver stage maturation and determined whether HSP101 is the limiting factor for the apparent paucity of secreted proteins. We generated and investigated transgenic *Plasmodium berghei* parasite lines that express fluorescent reporter proteins or HSP101 in liver stages, and, by genetic crosses, both simultaneously. We could show that reporter proteins, which are exported to the erythrocyte cytoplasm, fail to cross the membranes of the parasitophorous vacuole and tubovesicular network during liver stage development. For instance, *P. berghei* IBIS1 contains a conventional PEXEL motif that mediates export in *Plasmodium falciparum* blood stages (Petersen et al., 2015), but this is apparently not efficiently recognized for protein export in liver stages. Accordingly, any protein export occurring in the liver stages is not analogous to protein export in the blood infection stage.

We also analyzed an unconventional PEXEL sequence, of the sporozoite surface antigen CSP. However, when mCherry was linked to this motif the corresponding fusion protein remained confined to the PV of developing liver stages, although it was efficiently targeted to host erythrocytes during blood infection. Our data corroborate the notion that CSP is constantly shed during sporozoite transmigration, but not actively exported after conversion to liver stage development (Cockburn, et al., 2011), and refute the suggestion that CSP is exported to the host nucleus to reprogram the infected hepatocyte (Singh et al., 2007). As of now, LISP2 and SLTriP remain the only liver stage proteins reported to translocate during mid-liver stage development. Carboxy-terminal mCherry tagging of LISP2 leads to accumulation of mCherry in the PV, and it was suggested that carboxy-terminal processing of LISP2 is required for export (Orito, et al., 2013). Signatures of exported but PEXEL-negative proteins remain poorly understood (Spielmann and Gilberger 2010), and in perspective, discovery of liver stage-specific export signals might help to define the liver stage exportome.

In other pathogens, protein translocons fail to export larger, stably folded proteins, therefore limiting translocation. For instance, bacterial type III secretion systems fail to translocate stably folded fluorescent proteins (Akeda and Galan 2005). In this study, reporter constructs harbored a PEXEL linked to mCherry, which is a relatively packed and large (29 kDa) protein, potentially limiting export of the reporter constructs in the liver stage in spite of efficient export in the blood. However, the model antigen OVA can be translocated by bacterial type III secretion systems (Wiedig et al., 2005), and is also not detected outside the liver stage PV when fused to the region of CSP that drives protein translocation across the blood stage PVM. Tagging with fluorescent proteins does not appear to limit protein translocation in the blood, and export of the mCherry reporters we used in our study was consistently detected in asexual blood stages. Translocation of fluorescent proteins in the blood stage requires functional HSP101 for protein unfolding (Matthews et al., 2019b), so the lack of mCherry export in the liver in the absence of HSP101 is perhaps expected. However, we were able to demonstrate that expression of untagged HSP101 in the liver stage from the *PTEX150* promoter still did not allow liver-stage parasites to export the mCherry reporter constructs. Clearly, more work is warranted to decipher protein export by liver stage *Plasmodium*. To this end, smaller tags that permit immunodetection might further minimize constraints by protein folding.

*Plasmodium* is an obligate intracellular pathogen and has to avoid immune recognition inside the only nucleated host cell, the hepatocyte, during the first population expansion phase in the liver. Parasite replication inside a PV is a powerful shield to minimize antigen exposure at the surface of the infected cell. The corresponding trade-offs are nutrient depletion and waste accumulation. During blood infection extensive erythrocyte remodeling poses no danger, since these terminally differentiated host cells are devoid of MHC class I -dependent antigen presentation. Previous studies have proposed a concept of regional PV export protein storage in asexual blood stages (Bullen, et al., 2012). These export areas are proposed to localize to specific bulges in the PV, which are defined by PTEX assembly, which in turn might be initiated by EXP2 localization determined by host cell factors (McMillan et al., 2013; Meibalan et al., 2015; de Koning-Ward et al., 2016). Proteins resistant to unfolding get trapped in loop-like PVM extensions in blood stages, and unblocking the unfolding capacity only partially recovers protein export, as cargo remains spatially segregated from the export machinery (Charnaud et al., 2018). Although our results indicate localization of cargo proteins in the PV, previous studies propose that proteins need to remain in defined export zones for efficient translocation, which might be determined by yet unknown proteins. PV-localized accessory proteins are involved in the export of proteins in blood stages (Elsworth et al., 2016; Batinovic et al., 2017). Whether this complex system relies on further accessory proteins, thereby raising the question whether these proteins are present in liver stages, remains to be determined.

Proper PTEX assembly plays a major role in effective protein export. Despite introducing HSP101 expression in liver stages to facilitate PTEX assembly, the mCherry signal of reporter constructs were confined to the parasite PV and not detectable in the hepatocyte cytoplasm. Thus, we conclude that transgenic parasites were not able to recreate the PEXEL-dependent export machinery in liver stages. Although expression of all core components was induced in the transgenic parasites, we were unable to confirm proper assembly of the complex in liver stages. Carboxy-terminal truncation of PTEX150, the linker between the pore spanning EXP2 and the AAA^+^ ATPase HSP101, led to decrease in quantity of other PTEX components (Elsworth et al., 2016), suggesting complex destabilization. In this study, HSP101 was carboxy-terminally tagged, possibly interfering with correct protein folding. Indeed, recent structural analysis of PTEX complex assembly revealed stable binding of the EXP2 protomer to the carboxy-terminal ends of the HSP101 protomer, thereby placing the HSP101 cargo tunnel directly on the top of the EXP2-PTEX150 pore (Ho, et al., 2018). This suggests that interfering with the HSP101 carboxy-terminus, and as a consequence, with proper protein folding may prevent PTEX assembly; incomplete or unstable complex assembly has been proposed to inhibit protein translocon activity (de Koning-Ward, et al., 2016). Tagging HSP101 with mCherry has been shown to disrupt its function (Matthews et al., 2019b). Specifically, C-terminal mCherry tagging has been shown to interfere with unfolding of soluble translocated proteins and to interfere with parasite growth in the blood. Our findings of reduced growth of the *ef1α:HSP101-mCherry* are consistent with these findings. A smaller tag was well tolerated in this study (Matthews et al., 2019b). We note that we also expressed not only a myc-tagged HSP101, but also a native HSP101 protein in our promoter swap approach. Since these parasites displayed similar paucity of protein export, we consider dysfunctional PTEX assembly due to their carboxy-terminal tags less likely.

*In vitro* infection of hepatoma cells with *ef1α:HSP101-mCherry* parasites revealed HSP101 expression. However, the mCherry tag signal in the PV was weak, questioning if reconstruction of the machinery is feasible. As *HSP101:5′ptxe150* parasites lack a tagged version of HSP101, mRNA levels confirmed increased liver *HSP101* transcription compared to WT parasites, but whether HSP101 localizes to the PV remains to be determined. While we cannot rule out the possibility of post-translational repression, we believe this is unlikely to be the case because the regulatory regions used are well characterized, and visualization of *HSP101-mCherry* in early liver stages was possible in *ef1α:HSP101-mCherry* parasites*.* In contrast, endogenous *HSP101* is controlled at the transcriptional level in this stage; however, low mRNA levels were detectable in WT parasites, whereas HSP101 protein is absent in early liver stages (Matz et al., 2015), raising the questions whether endogenous *HSP101* is post-transcriptionally repressed.

It remains to be determined if further, yet unidentified, parasite and host proteins can contribute to protein export in liver stages. The core components EXP2, PTEX150 and HSP101 are essential for PTEX assembly and blood stage parasite survival (de Koning-Ward et al., 2009; Matthews et al., 2013; Matz et al., 2013). Previous work established that decreased HSP101 levels blocked export and led to vesicular accumulation of proteins in blood stages (Beck et al., 2014; Elsworth et al., 2014). Ho et al. indicate that the stoichiometry of 6:7:7 for HSP101-PTEX150-EXP2 is a prerequisite for functional PTEX assembly (Ho et al., 2018). Presently, it remains unknown whether the *ef1α* promoter or the promoter swap with the *ptex150* promoter yields sufficient protein. This can be experimentally tested by placing *HSP101* under the control of a strong pre-erythrocytic promoter.

*In vivo* infections demonstrated half a day delay in pre-patency for *ef1α:HSP101-mCherry* and *HSP101:5′ptex150*. Similarly, subsequent blood stage growth was delayed and parasitemia remained lower, however slope analysis demonstrated no significant differences in growth rate. Thus, it can be postulated that intra-hepatic growth is slightly affected, whereas blood stage development remains unaffected by *HSP101* overexpression. This notion is strengthened by parasite loads of infected mouse livers, which were slightly elevated at 48 h after infection in *ef1α:HSP101-mCherry* compared to WT -infected cohorts. As budding of merozoite-filled merosomes initiates as early as 48 h after infection in WT parasites (Sturm et al., 2006), slightly elevated parasite loads in *ef1α:HSP101-mCherry* infected mice could indicate a delay in maturation and budding of merosomes leading to the observed delay in pre-patency. Whether this is actually caused by *HSP101* overexpression itself or subsequent up- or downregulation or obstruction of other liver stage proteins remains to be determined.

To address PEXEL-dependent export in liver stages it is of interest to determine PTEX assembly with the additional HSP101 copy in engineered parasites. Lack of *P. berghei-*specific PTEX component antibodies prevented confirmation of PTEX assembly with immunoprecipitation. However, despite the lack of PTEX assembly verification, we observed localization of HSP101 to the PV of the parasite in liver stages and could confirm elevated *HSP101* mRNA levels in early liver stages. Additionally, PTEX assembly is vital for parasite survival in blood stages, therefore, a functional PTEX complex and correct HSP101 positioning in the complex is present in the promoter swap parasites *HSP101:5′ptex*. Localization of the tagged HSP101 copy in *ef1a:HSP101-mCherry* parasites was observed in the cytoplasm and a more prominent stain in the PV of the parasite. We note that the bulky mCherry tag could interfere with the correct localization of HSP101. This might affect PTEX assembly in parasites harboring an additional copy of HSP101.

Taking advantage of pathogen stage conversion for immunization strategies is one way to attack the parasite at its developmental bottleneck (Kreutzfeld et al., 2017). Studies have shown that superior immunity can be achieved by a developmental arrest shortly before liver-to-blood stage conversion, as a larger and broader array of antigens is presented compared to early arresting GAPs (Butler et al., 2011; Haussig et al., 2011). Therefore, we performed immunizations with azithromycin attenuated late arresting liver stages and challenged vaccinated animals to explore whether restoring HSP101 in liver stages can elicit superior immune responses. We, however, detected inferior protection, and whether this can be attributed to alteration of protein export during liver stage development needs to be explored further.

In conclusion, a functional PTEX complex, as seen in infected erythrocytes, cannot be reconstructed solely by ectopic HSP101. Our work also highlights that liver stage-host cell interactions remain relatively ill defined. The tight control of protein export during liver development likely contributes to poor recognition and cytolysis by the host immune system. Ultimately, identifying candidate liver stage-specific protein export signals, defining the contribution of PTEX in liver stage protein export, and characterizing the liver stage exportome by proteomic approaches are key to a better mechanistic understanding of immunity and vaccine development against *Plasmodium* pre-erythrocytic stages.

## Methods

### Ethics Statement

All animal work was conducted in accordance with the German “Tierschutzgesetz in der Fassung vom 18. Mai 2006 (BGBl. I S. 1207)”, which implements the directive 86/609/EEC from the European Union and the European Convention for the protection of vertebrate animals used for experimental and other scientific purposes. The ethics committee of MPI-IB and the Berlin state authorities (LAGeSo Reg# G0469/09 and G0294/15) approved the protocol.

### Parasites and Experimental Animals

*P. berghei* ANKA cl507 parasites that constitutively express GFP under the control of the *EF1α* promoter (Franke-Fayard et al., 2004) and *P. berghei* ANKA bergreen parasites that constitutively express GFP under the control of the *HSP70* promoter (Matz et al., 2013) were used in our experiments. 6–8 weeks old female NMRI or C57BL/6 mice used in this study were either purchased from Charles River Laboratories, Janvier, or bred in-house. NMRI mice were used for transfection experiments, blood stage infections and transmission to *Anopheles stephensi* mosquitos. C57BL/6 mice were used for sporozoite infections and subsequent immunological studies.

### Generation of Transfection Vectors

To express an additional copy of *PbHSP101* (PbANKA_0931200) under a constitutive promoter the expression cassette was inserted into a silent locus on chromosome 6, using the pBART-SIL6 vector (Kooij et al., 2012). This plasmid harbors a drug selectable *hDHFR-yFcu* cassette for positive/negative selection, a GFP under the *PbHSP70* promoter for parasite life cycle analysis, an mCherry/triple c-Myc (3xMyc) tag sequence for protein tagging under the control of the 3′ region of *PbPPPK-DHPS* for mRNA stability, and two homologous sequences for stable integration into the silent locus on chromosome 6. The *HSP101* open reading frame was amplified using primers HSP101for and HSP101rev (Supplementary Table S2). The fragment was inserted *via Xba*I and *Age*I 5′ to the mCherry/3xMyc tag. For the plasmids *EF1α:HSP101-mCherry* and *EF1α:HSP101-myc* the 5′ region of *EF1α* (PbANKA_113330) was amplified using primers EF1for and EF1rev (Supplementary Table S2). The fragment was inserted *via BssH*II and *Xba*I 5′ next to the *HSP101* open reading frame. mCherry was excised in the plasmids *EF1α:HSP101-mCherry*, and replaced by a linker sequence, generated with Linkfor und Linkrev (Supplementary Table S2), fusing the *HSP101* open reading frame and the 3xMyc tag (Supplementary Table S2). For swap of the endogenous promoter with the 5′ region of *PTEX150* a pBART-based plasmid containing the *PTEX150* promoter linking *HSP101* ORF was generated. In brief, the amino-terminal portion of the *HSP101* ORF (primers HSP101_amino_F, HSP101_amino_R in Supplementary Table S2) was integrated under the control of *PTEX150* promoter (primers PTEX150-5′-PrimerF, PTEX150-5′-PrimerR in Supplementary Table S2) integrated with *Sac*II and *Hpa*I. The 5′UTR of HSP101 amplified with HSP101-5′-F and HSP101-5′-R (Supplementary Table S2). The 5′UTR together with the *HSP101* amino-terminus served as homologous recombination sites (Supplementary Figure S3).

The mCherry-based export reporters were constructed in the B3D+mCherry vector (Silvie et al., 2008b). The sequence encoding the first 70 amino acids of *CSP* was amplified with CSPfor and CSP_70_rev (Supplementary Table S2) and cloned into the *Xba*I and *Spe*I sites of B3D+mCherry, and the 5′ genomic regions directly upstream of *UIS4*, *HSP70* and *IBIS1* were amplified with UIS4-5′for and UIS4-5′rev, HSP70-5′for and HSP70-5′rev, and IBIS1-5′for and IBIS1-5′rev (Supplementary Table S2), respectively, and inserted into the *Sac*II and *Not*I sites. Plasmids were linearized with *Bsm*I, *Hpa*I or PacI, respectively, prior to transfection. IBIS1_1-90_ and IBIS1_1-118_ were amplified together with the *IBIS1* 5′ region using primers IBIS1-5′for and IBIS1_90_rev or IBIS1_118_rev (Supplementary Table S2), respectively and inserted into the *Sac*II and *Spe*I sites of B3D + mCherry (Supplementary Figure S2). The IBIS1 plasmids were linearized with *Pac*I prior to transfection.

### Generation of Transgenic Parasites

In order to generate stable transgenic parasite lines, NMRI mice were infected with 100 μl thawed cryopreserved *P.b.* ANKA blood stage parasites peritoneal and parasites were grown until a parasitemia of ∼3–5%. Parasite culture and schizont isolation were done as described (Janse et al., 2006). Schizonts were transfected with 5–10 μg *Apa*LI linearized plasmids using the AMAXA Nucleofector device (program U33). Electroporated parasites were mixed with additional 50 μl transfection medium and intravenously injected into NMRI mice. The linearized *ef1α:HSP101-mCherry* and *ef1α:HSP101-myc* plasmids integrated in the silent locus in chromosome 6 (Supplementary Figure S3), and positive recombination events were selected with pyrimethamine (70 mg/L) in the drinking water, followed by FACS of GFP-positive iRBCs to generate isogenic transgenic parasite lines. The linearized *hsp101:5′ptex150* plasmid integrated between the endogenous *HSP101* 5′UTR and ORF resulting in a new, constitutively active promoter for *HSP101* expression (Supplementary Figure S3C). Recombinant *hsp101:5′ptex150* were selected by pyrimethamine, and an isogenic line was established by flowcytometric (FACS) cloning as described (Kenthirapalan et al., 2012). To verify successful integration, transgenic parasites DNA was extracted from blood stage cultures, and genotyping was performed with oligonucleotides specific for 5′ and 3′ integration (Supplementary Table S2) as well as for the WT locus (Supplementary Table S2, Supplementary Figure S3B+D).

### Immunofluorescence Staining and Live Imaging of Infected Red Blood Cells

Parasite blood stages were visualized in immunofluorescence assays (IFA). Infected RBCs were retrieved by tail vein punctures of infected mice, mixed with 1x PBS and placed on a poly-l-lysine covered 12 mm ø cover slips in a 24-well plate to settle. Cells were fixed with 4% PFA/0.0075% fresh glutaraldehyde (GA) at RT. After three washing steps cells were permeabilized with 0.2% Trition X-100 (in PBS) for 20 min at RT. Cells were washed and blocked with 3% BSA, 0.2% Triton X-100 (in PBS) for 30 min at RT on a shaker. Primary antibody was added in blocking solution for 1 hour at RT, followed by three washing steps and incubation with fluorophore labeled secondary antibody and Hoechst 33,342 in blocking solution for 1 h at RT. Additional washing steps removed remaining secondary antibody before cells were mounted with Fluoromount G on a glass slide. Live imaging of iRBC was performed to access expression of mCherry and GFP as well as adequate growth of the parasites. iRBCs were placed on a glass slide and covered with a cover slip. Cells were analyzed with an Zeiss Axio Observer immunofluorescence microscope, and images were taken at 630x magnification.

### Salivary Gland Sporozoite Isolation

WT GFP-expressing *P. berghei* ANKA and transgenic strains were maintained by continuous cycling in NMRI mice and female *Anopheles stephensi* mosquitoes (Vanderberg, 1975). Mosquitoes were kept at 28°C (non-infected) or 20°C (infected) at 80% humidity. 10–14 days after mosquito infection, midguts were isolated and oocyst development analyzed by fluorescent microscopy. Salivary gland sporozoites were isolated on day 17 post infection from mosquitos in DMEM medium containing 10% fetal calf serum (FCS).

### Immunofluorescence Staining of Salivary Gland Sporozoites

Salivary gland sporozoites were visualized by immunofluorescence assay (IFA). Briefly, 10,000 salivary gland sporozoites suspended in 3% BSA-RPMI were added into each ring of a Medco glass slide pre-coated with 3% BSA-RPMI and incubated for 15 min at 37°C. Slides were fixed with 4% paraformaldehyde (PFA), permeabilized with 0.2% TritonX-100 and blocked with 3% BSA. Sporozoites were stained with chicken anti-GFP antibody, followed by anti-chicken Alexa Fluor 488-coupled antibody (Invitrogen, OR, United States), rat anti mCherry antibody followed by anti-rat Alexa Fluor 546-coupled antibody (Invitrogen, OR, United States) and the nuclear stain Hoechst 33,342 (1:5,000; Invitrogen, OR, United States). Slides were mounted with Fluoromount-G (SouthernBiotech) prior to analysis by fluorescence microscopy using a Zeiss Axio Observer immunofluorescence microscope, and images were taken at 630x magnification.

### Parasite Infectivity *in vivo*

For analysis of pre-patency and blood stage growth after sporozoite infection, 10,000 WT or transgenic sporozoites were injected intravenously into to C57BL/6 mice (*n* = 5 each). Tail punctures for blood smears were performed daily from day 3 after infection onwards until day 14. Smears were stained with a 10% Giemsa solution and analyzed by microscopic examination for presence and percentage of blood stage parasites. For natural mosquito infections 15 infected mosquitoes were kept in a separate container allowing a targeted blood meal on one C57BL/6 mouse. Mice were anesthetized with ketamine/xylazine intraperitoneal (i.p.), and mosquitoes were allowed to feed for 15 min. Mosquitoes were analyzed for successful feeding by examining blood uptake in the mosquito midgut.

### Quantification of Parasite Burden in Mouse Livers

Groups of 5 C57BL/6 mice were infected with 10,000 WT or *EF1α:HSP101* sporozoites each, and the livers were harvested 42 h later. Livers were homogenized in TriZol (Thermo Fischer), and total RNA was extracted. cDNA was generated by reverse transcription with the RETROScript Kit (Ambion). Quantitative real-time PCR (qPCR) analysis was done with primers specific for *Pb*18S rRNA and mouse *GAPDH* for normalization (Supplementary Table S2). Relative liver parasite levels were measured applying the ∆∆Ct method.

### Crossing of Recombinant Parasite Lines

For crossing two parasite lines in the mosquito, female NMRI mice were infected intravenously (i.v.) with the parasite lines. On day 4, parasitemia was determined and five million parasites of each line were intravenously injected into one female NMRI mouse for subsequent mosquito infection. After confirmation of exflagellation of male gametes mosquitoes were allowed to take a blood meal on the infected mouse.

### Hepatoma Cell Infection *in vitro* and Immunofluorescence Staining

Cell lines were kept according to standard cell culture conditions for human hepatoma cell lines (Huh7). In brief, Huh7 cells were cultured in DMEM complete (DMEM, 10% FCS, 1% Penicillin-Streptomycin) at 37°C and 5% CO2. Nunc Lab-Tek II 8-well Chamber Slides (ThermoFischer, NY, United States) were seeded with 30,000 Huh7 cells per well 24 h before infection. 10,000 WT or transgenic salivary gland sporozoites suspended in 100 μl DMEM-complete were added, centrifuged and allowed to settle for 2 h at RT. Cells were washed 2 h later to remove non-invaded sporozoites, and then medium was changed daily. Growth was terminated at 2, 16, 24, 40, 48, 54 and 72 h after infection by fixation with 4% PFA for 10 min at RT. Infected cells were blocked with PBS/10%FCS and permeabilized with 0.2% Triton (Roth, Karlsruhe, Germany) for 1 h at 37 °C. Primary antibodies (*α*-GFP, *α*-USI4, *α*-mCherry, *α*-Myc, *α*-*Pb*HSP70, and *α*-CSP) were added for 1 hour at 37 °C. After washing, cells were incubated with the respective fluorescently labeled secondary antibody (*α*-chicken Alexa Fluor 488, *α*-mouse Alexa Fluor 546, *α*-mouse Alexa Fluor 488, *α*-rat Alexa Fluor 546, *α*-rat Alexa Fluor 488, *α*-rabbit Alexa Fluor 546, *α*-rabbit Alexa Fluor 488; Invitrogen, OR, United States) and Hoechst 33,342 (Invitrogen, OR, United States) for 1 hour at 37 °C. Subsequently, cells were washed and mounted with Fluoromount G (SouthernBiotech) and imaged by fluorescence microscopy (Zeiss Axio Imager Z2) with 630x magnification.

### Live Imaging of Infected Hepatoma Cells

For protein export analysis of infected Huh7 cells with crossed transgenic parasite lines, live imaging of parasites was performed. Infected cells were cultured in Ibidi slides, washed once with PBS and remained in PBS solution for the time of imaging. Live imaging was performed with a Zeiss Axio Observer immunofluorescence microscope with 1,000 magnification. Duration of analysis was kept at a minimum to ensure cell survival.

### Quantification of mRNA Levels in Liver Stages

To determine expression of liver stage specific genes, relative mRNA levels were determined in WT and transgenic parasites. 300,000 Huh7 cells/well were plated in a 24-well plate. One day later, salivary gland sporozoites were isolated from infected mosquitoes and Huh7 cells were infected with 100,000 sporozoites/well. For parasite sedimentation plates were centrifuged for 10 min at 3,000 rpm, no brake, and incubated at 37 °C and 5% CO_2_. Two hours later, cells were washed once to remove extracellular sporozoites. After 16, 24, 48 or 72 h cells were trypsinized with 200 μl for 3 min at 37 °C, and carefully resuspended in DMEM and washed with additional DMEM. After 5 min centrifugation at 1200 rpm with brake, the pellet was resuspended in 500 μl TRIzol, and RNA was extracted for subsequent cDNA synthesis and qPCR analysis as described previously. qPCR analysis was performed with *HSP101* and *HSP70* primers (Supplementary Table S2).

### Immunization With Attenuated WT and Transgenic Sporozoites

To examine vaccine efficacy mice were immunized mice in a prime boost regimen (day 0 and day 7) with 10,000 sporozoites delivered i. v. Salivary gland sporozoites were attenuated either by a *γ*-irradiation dose of 12 × 10^4^ cGy, or with azithromycin (AZ) cover (4.8 mg in 200 μl of sodium chloride solution per mouse) (Friesen et al., 2010). For challenge experiments, immunized and control mice were i.v. injected with 10,000 WT *P. berghei* ANKA sporozoites 87 or 260 days post immunization. All mice were checked for parasitemia by microscopical examination of Giemsa-stained blood smears starting at day 3 after challenge inoculation until day 14. Animals that remained parasitemia-free were continuously checked for parasitemia up till 45 days after the challenge.

### Statistics

Statistical significance was analyzed by non-parametrical Whitney-U test for non-normally distributed data (infected hepatoma cells) using GraphPad Prism V5.0c. Log rank (Mantel-Cox) test was applied for Kaplan-Meier analyses, and slope of parasite growth was analyzed using R (RStudio version 0.99.879).

## Data Availability

The original contributions presented in the study are included in the article/Supplementary Materials, further inquiries can be directed to the corresponding author.
