# Supplementary material for: Absence of PEXEL-Dependent Protein Export in Plasmodium Liver Stages Cannot Be Restored by Gain of the HSP101 Protein Translocon ATPase
Source: Front Genet. 2021 Dec 8;12:742153. doi: 10.3389/fgene.2021.742153 (PMC8693896; doi:10.3389/fgene.2021.742153)
Supplement: Supplementary file 1 [file DataSheet1.pdf]

Supplementary Material

Absence of PEXEL-dependent protein export in *Plasmodium* liver stages cannot be restored by gain of the HSP101 protein translocon ATPase

Oriana Kreutzfeld, Josephine Grützke, Alyssa Ingmundson, Katja Müller, Kai Matuschewski

Contents:

- **Figures S1 - S6**
- **Tables S1-S2**

Kreutzfeld *et al.*, Figure S1

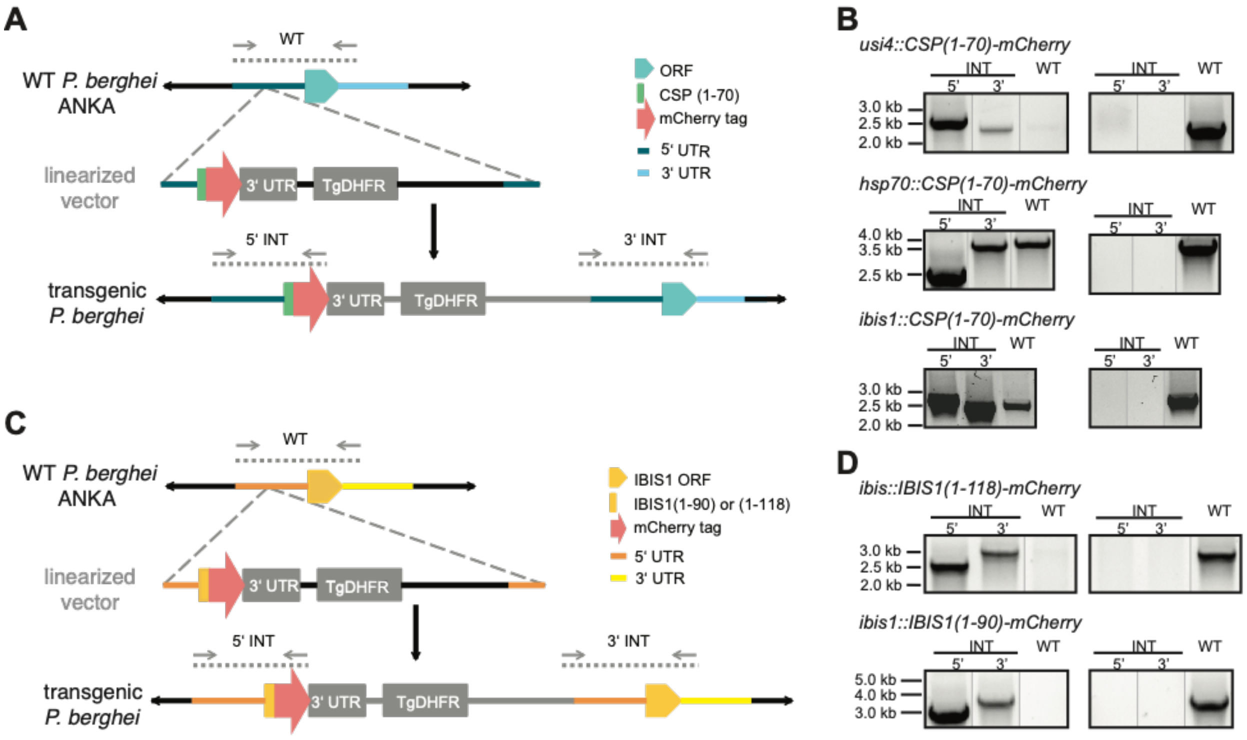

Supplementary Figure 1. Generation of reporter parasite lines.

Shown are the (A,C) recombination events and (B,D) diagnostic PCRs to confirm the desired integrations.

(A,C) Integration strategy for the (A) CSP$_{1-70}$-mCherry and (C) IBIS$_{1-118}$ reporter proteins. Shown are the WT locus (top), the linearized targeting plasmid (centre) and the recombinant locus (bottom). Diagnostic primers and PCR products are shown as arrows and dotted lines.

(B,D) Diagnostic PCRs show successful integration of the targeting constructs in the recombinant parasites (left) in comparison to WT parasites (right) with the PCR products, as depicted in the schemes.

Kreutzfeld *et al.*, Figure S2

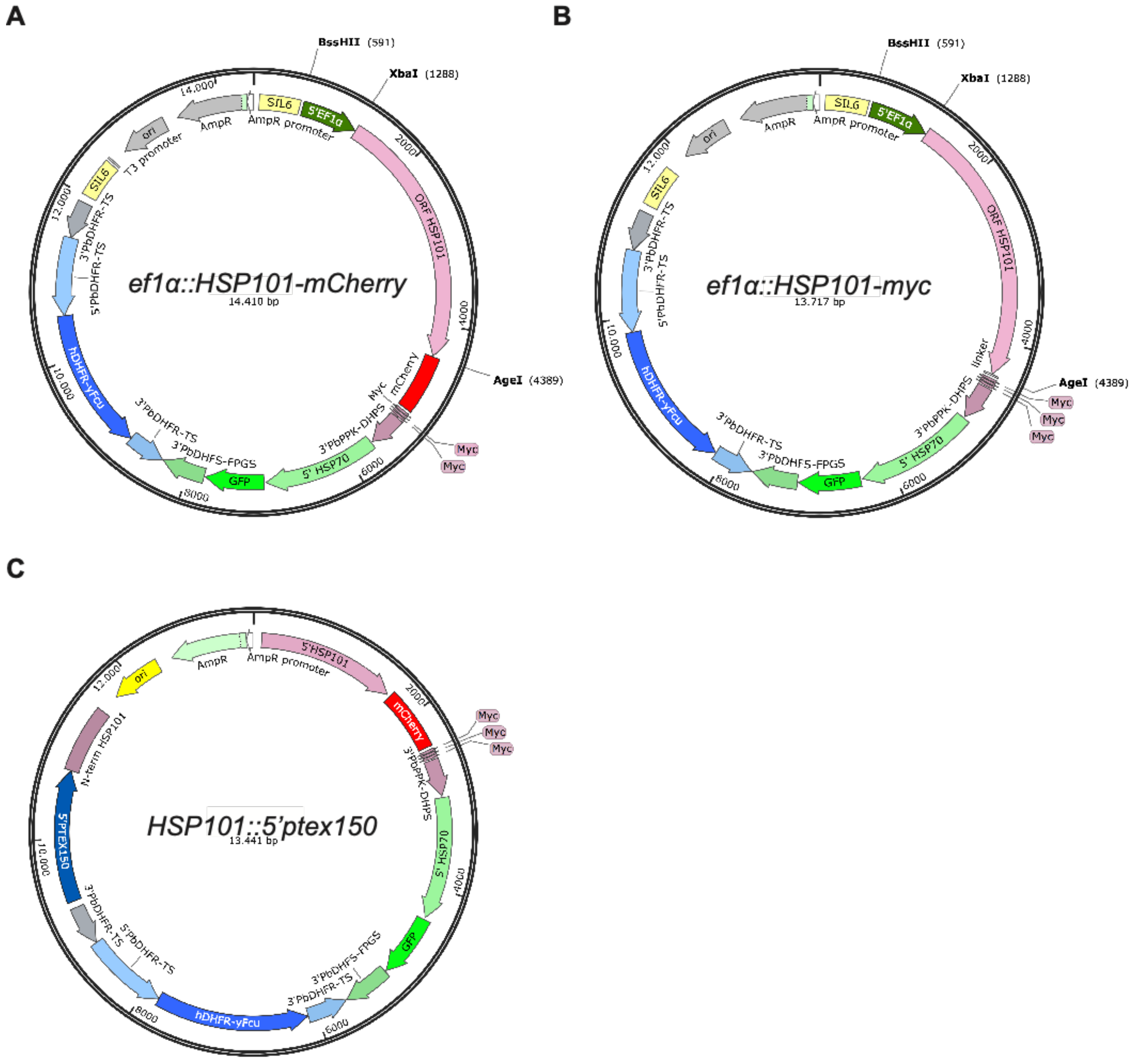

Supplementary Figure 2. Targeting vectors to generate recombinant *HSP101*-expressing parasite lines.

Shown are the vector maps with the genetic elements highlighted in colors for the (A) *ef1α::HSP101-mCherry,* (B) *ef1α::HSP101-myc* and (C) *HSP101::5'ptex150* targeting vectors.

Kreutzfeld *et al.*, Figure S3

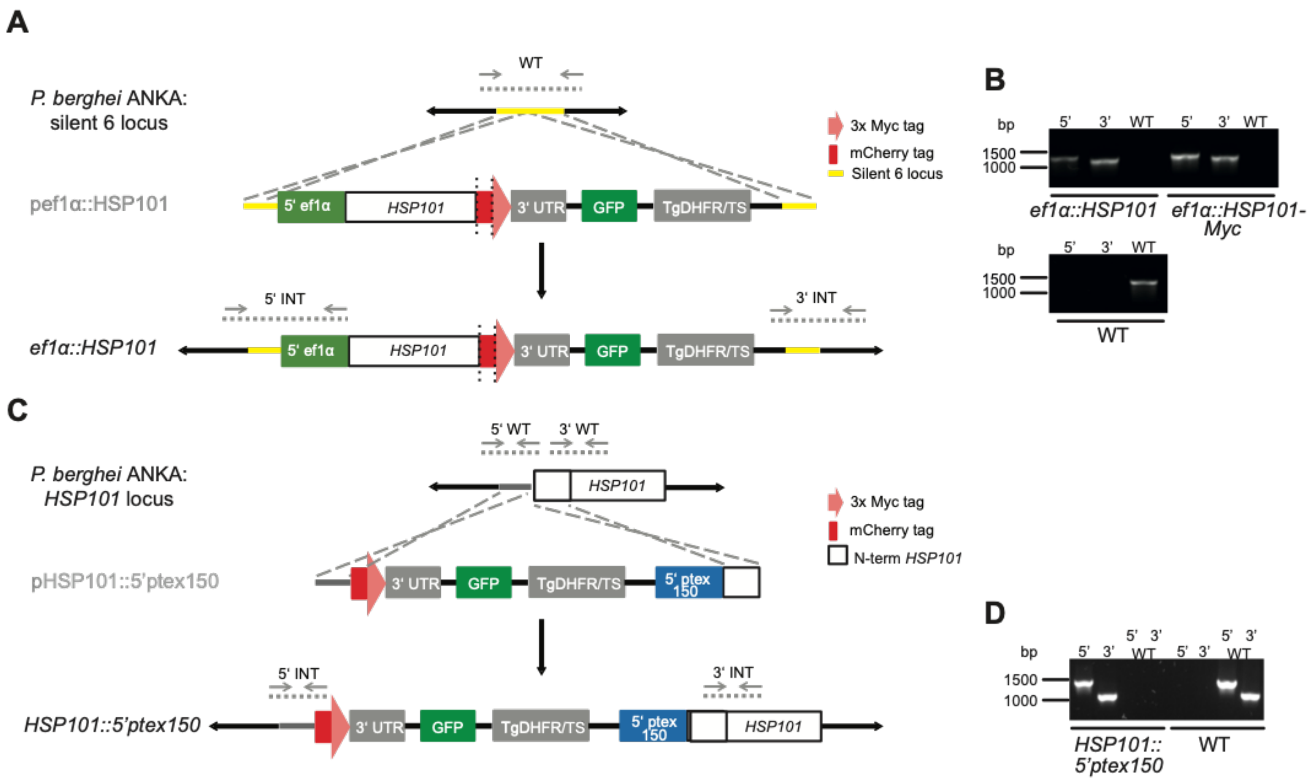

Supplementary Figure 3. Generation of transgenic *HSP101* parasite lines.

Shown are the (A,C) recombination events and (B,D) diagnostic PCRs to confirm the desired integrations.

(A,C) Integration strategy for the (A) *ef1α::HSP101* and (C) *HSP101::5'ptex150* parasite lines. Shown are the WT locus (top), the linearized targeting plasmid (centre) and the recombinant locus (bottom). Diagnostic primers and PCR products are shown as arrows and dotted lines.

(B,D) Diagnostic PCRs show successful integration of the targeting constructs in the recombinant parasites in comparison to WT parasites with the PCR products, as depicted in the schemes.

Kreutzfeld *et al.*, Figure S4

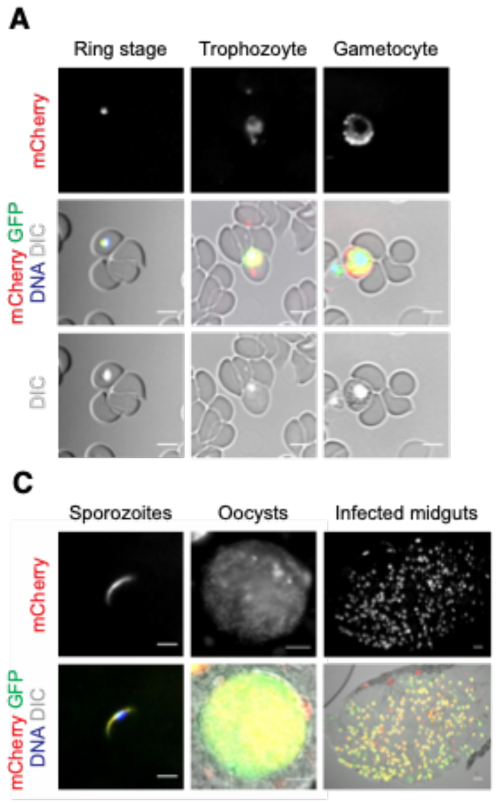

Supplementary Figure 4. Live imaging of *ef1α::HSP101 parasites*.

(A) Asexual (ring stage, trophozoite) and sexual (gametocyte) *ef1α::HSP101-mCherry* blood stages from the peripheral blood. Shown are the mCherry signal (top) and merge images (bottom) of mCherry (red), GFP (green), Hoechst 33342 (blue), and DIC images. Bar, 5 µm.

(B) Asexual (ring stage) and sexual (gametocyte) *ef1α::HSP101-myc* blood stages from peripheral blood. Shown are the GFP and Hoechst 33342 signal (top) and merge images (bottom), including DIC images. Bar, 5 µm.

(C) Sporozoites, oocysts and infected midguts of *ef1α::HSP101-mCherry* parasites. Shown are the mCherry signal (top) and merge images (bottom) of mCherry (red), GFP (green), Hoechst 33342 (blue), and DIC images. Bar, 5 µm.

Kreutzfeld *et al.*, Figure S5

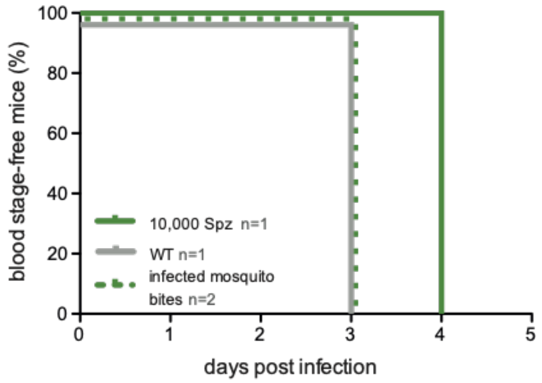

Supplementary Figure 5. Sporozoite-induced blood infection in *ef1α::HSP101-myc* parasites.

Shown in an exploratory Kaplan-Meier analysis of blood infection over time after intravenous injection of 10,000 sporozoites or exposure to 15 infected mosquitoes. Blood infection was monitored daily by microscopic examination of Giemsa-stained blood films.

Kreutzfeld *et al.*, Figure S6

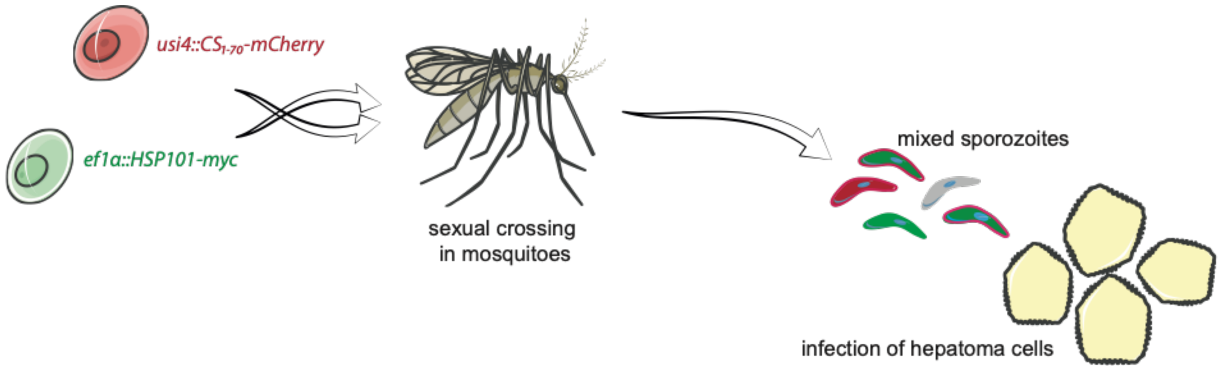

Supplementary Figure 6. Genetic crosses by sexual recombination in *Anopheles stephensi* mosquitoes.

Schematic representation exemplified by coinfection of *ef1α::HSP101-myc* and *UIS4::CSP*$_{1-70}$-*mCherry* parasites. The progeny was used for hepatoma cell infections and double fluorescent parasites were imaged to monitor protein secretion during liver stage development. Illustrations modified and adapted from Smart Servier Medical Art (Servier).

Supplementary Table 1. Midgut oocysts and salivary gland sporozoites from *Anopheles Stephens* mosquitoes infected with WT and transgenic parasite lines

Parasite	% infectivity (d10 midgut oocysts)[a]	# salivary gland sporozoites per mosquito[b]
WT	47 %	17,500 (±15,600)
ef1α::HSP101-mCherry	50 %	14,300 (± 9,900)
ef1α::HSP101-myc	73 %	11,750 [c]
HSP101::5'ptex150	70 %	18,400 (± 4,600)

[a] Mean number from three independent experiments

[b] Mean number from six independent experiments

[c] Data from a single experiment only

Supplementary Table 2. Primer sequences for generations of plasmids, genotyping and qRT-PCR.

Name	Sequence *
CSPfor	GCATCTAGAAAAGGTAAAATGAAGAAGT
CSP_{70} rev	CGGACTAGTCCGATCGGCAAGTA
UIS4-5'for	TGACCGCGGCTTTATGTTTTTGAAAGATTTAAC
UIS4-5'rev	GATGCGGCCGCTTTATTCAGACGTAATAAT
HSP70-5'for	TGTCCGCGGCATGATTTATTTATTTGC
HSP70-5'rev	GATGCGGCCGCCTTTTTTTAATTGTAATTG
IBIS1-5'for	GCATCCGCGGCGTATTTAATCATACACTATACGTTTTTCC
IBIS1-5'rev	CGGGCGGCCGCTTTTTAACTAATATGTTTTC
$IBIS1_{90}$rev	GGACTAGTATCCAACTCTGATAATATTCTGCTTTTTCC
$IBIS1_{118}$rev	GGACTAGTTGACATATATGAGGTTGATGCATTTTCAG
5' int B3D rev	GATCCTTACTTGTACAGC
3' int B3D fwd	CTAATACGACTCACTATAGGGC
HSP70 5'int fwd	CCATAATGTTCGATCGAGCAAATAACTTT
HSP70 3'int rev	CGACTCCATTCACTTTATTCCTTTTTCAT
UIS4 5'int fwd	CAAATAATTGTGTATGTAATATTTAAGTGTAGAATGGAAT
UIS4 3'int rev	CTCTATAACATTATTTTTCCTTTTCTCTTACTT
IBIS1 5'int fwd	AAAATAATACATTTAATGAAGGGGAAAAGG
IBIS1 3'int rev	CATTAAAATATACAATTTCGATATAATATGTTGATC
HSP101for	CATCTAGAATGGTACGGAACATTGCTAAAAATTATTTATTTG
HSP101rev	GCCCTTGCTCACACCGGTTGACAATGAAAGGTTAATAACAATGTTG
5'EF1a fwd	CATGAGCGCGCGATTTACATGGCGTTTTATGTTTATATG
5'EF1a rev	GCTCATCTAGATTTTATAAAATTTTTATTTATTTATAAGCAAATATA
linker fwd	CGAGTGGCCACGTGGCCACCAGAACCACCACCGGTTCATG
linker rev	CATGAACCGGTGGTGGTTCTGGTGGCCACGTGGCCACTCG
HSP101-5'-F	AGTACCCCGCGGATAAATAGAATAAGATGCTTGCTTTCG
HSP101-5'-R	ATCAGGGTTAACTAAATTTATAGTAAATATAGATATAATTTTATCTTCATTC
HSP101_amino_F	CCCGGGGTTTAAACATGGTACGGAACATTGCTAAAAAT
HSP101_amino_R	CCAAAGTTTTAGCTATTACCGC
PTEX150-5'-PrimerF	TTGTCTCCGCGGTATATAAAGTGTTAATAGTGTTTTTTTTTGTGC

PTEX150-5'-PrimerR	CTTGCCGTTAACTTTATTATTCTAATTTATTTATATTTTCGTTTCTTTTTG
WT fwd	GACAGCGCATATGATGGATG
WT rev	TTTGAGAAATTGCGTATTCGTA
5' integration fwd ef1a::hsp101	ATGAAATACCGCTCCATTTTTCC
3' integration rev ef1a::hsp101	CATCTAGATTTTATAAAATTTTTATTTATTTATAAGCAAATATA
3' integration fwd hsp101::5'ptex150	GACTGAGGTTGTGTGATGGC
3' integration rev hsp101::5'ptex150	GAGCCAAATTGTTCAATGTTTAAT
5' integration fwd hsp101::5'ptex150	CTATAAATTTAGTAGAAATGGTACGGAAC
5' integration rev hsp101::5'ptex150	GACACTTATATATTTATACTAAGTTCG
HSP70 fwd qPCR	GCTAACGCAAAAGCAAAGC
HSP70 rev qPCR	TCGGTAAAAGCTACATAGGATG
HSP101fwd qPCR	TTTGTTATTGTCTGTGCTCAAGG
HSP101 rev qPCR	GCAAAGGTTTTAATTGGTTATGACC
Pb18 fwd	AAGCATTAAATAAAGCGAATACATCCTTAC
Pb18 rev	GGAGATTGGTTTTGACGTTTATGTG
mGAPDH fwd	CGTCCCGTAGACAAAATGGT
mGAPDH rev	TTGATGGCAACAATCTCCAC

* Restriction sites are underlined
